# Association of Adult-Onset Bartter Syndrome With Undifferentiated Connective Tissue Disorder

**DOI:** 10.7759/cureus.17140

**Published:** 2021-08-13

**Authors:** Nida Saleem, Humaira Nasir, Danyal Hassan, Momena Manzoor

**Affiliations:** 1 Internal Medicine/Nephrology, Shifa International Hospital, Islamabad, PAK; 2 Histopathology, Shifa International Hospital, Islamabad, PAK; 3 internal Medicine/Nephrology, Shifa International Hospital, Islamabad, PAK

**Keywords:** bartter syndrome, hypochloremic metabolic acidosis, undifferentitated connective tissue disorder (uctd), juxta glomerular hyperplasia (jg hyperplasia), hypokalemia

## Abstract

Bartter syndrome is a rare autosomal recessive, salt-losing disorder characterized by hypokalemic hypochloremic metabolic alkalosis. We are reporting a case of 21 years old patient, who presented with lower limb weakness, marked hypokalemia, proteinuria, and renal impairment detected on laboratory evaluation. The diagnosis of Bartter syndrome was suspected by marked hypokalemia and was supported by renal biopsy which showed evidence of Juxtaglomerular (JG) hyperplasia. This is the first case report about clinicopathological features of the patient with acquired Bartter syndrome and associated undifferentiated connective tissue disorder manifesting as hypokalemia with paralysis.

## Introduction

Bartter syndrome is a disorder that has heterogeneous clinical manifestations like polyuria, polydipsia, salt craving, fatigue, and failure to thrive. It is a disorder of loop of Henle with excessive urinary losses of sodium, chloride, and potassium. The typically associated laboratory abnormalities include marked hypokalemia, metabolic alkalosis, and volume contraction leading to hyper-reninemia and hyperaldosteronism [1]. It was first described by Bartter et al. in 1962 [2]. This condition is usually diagnosed on antenatal examination or in early childhood, and its presentation in adults is very rare. Bartter syndrome can be inherited or acquired. Inherited Bartter syndrome is divided into five subtypes: types I-IV are due to a loss of function mutations and type 5 due to gain of function mutation. Types 1, 2, and 4 are usually called antenatal Bartter syndrome while type III is called adult-onset/classical Bartter syndrome. Type V Bartter syndrome can be distinguished from all other types by the presence of hypocalcemia and hypomagnesemia. In addition to this, there are several acquired causes of Bartter syndrome, including autoimmune disorders like Sjogren syndrome [3,4], Hashimoto thyroiditis [5], scleroderma, and several drugs like aminoglycosides, loop diuretics, amphotericin, etc.

## Case presentation

The patient, the 21-year-old male, resident of Kabul, Afghanistan, student of undergraduate but then left studies one year back due to ill health, was referred to our institution, with a history of documented 10 kg weight loss in 15 months, off and on episodes of vomiting, fever, arthralgia and an episode of progressive bilateral lower limb weakness for the last eight months. This weakness was associated with the history of proximal myopathy for the last eight months. In addition to this, there was no history of excessive intake of diuretics and laxatives. Besides this, his family history was insignificant for any renal disease.

On examination, his blood pressure was 85/60 mmHg, pulse 88/min and he was afebrile. His BMI was 20 kg/m^2^. Neurological examination showed no abnormalities except for reduced muscle tone, diminished lower limb reflexes, and 3/5 power in both lower limbs. The rest of the systemic examination was unremarkable. His ECG was normal.

Table 1 shows the detailed investigations of this patient after admission.

**Table 1:** Baseline laboratory workup U/L: units/L, TLC: total leukocyte count, BUN: blood urea nitrogen, ALT: alanine transaminase, AST: aspartate transaminase, GGT: gamma-glutamyl transferase, WBC: white blood count, RBC: red blood count, HPF: hematopoietic-promoting factor.

TLC (cells/cmm)	15.96 (neutrophils:79% lymphocytes: 14%)	Serum magnesium (mEq/L)	2.37
Hemoglobin (g/dL)	13.7	Serum phosphorous (mg/dL)	1.4
Platelet count (cells/µL)	575,000	Blood glucose random (mg/dL)	187
Serum creatinine (mg/dL)	3	AST (U/L)	22
BUN (mg/dL)	26	ALT (U/L)	22
Serum bicarbonate (mEq/L)	24	ALP (U/L)	144
Serum chloride (mEq/L)	90	Gama GGT (U/L)	31
Serum sodium (mEq/L)	126	Total bilirubin (mg/dL)	0.4
Serum potassium (mEq/L)	1.7	Direct bilirubin (mg/dL)	0.17
Serum calcium (mg/dL)	9.1	Urine R/E	Protein: ++, blood: +, 6-8 WBCs /HPF, 8-10 RBCs/HPF, urine PH: 7, urine specific gravity: 1.010
Urine protein to creatinine ratio (g/g)	1.3	Serum anion gap	12

Table 1 shows low levels of serum potassium, serum sodium, and serum phosphorous. Serum creatinine is also derranged. There is proteinuria, hematuria, leukocyturia, and alkalotic pH on urine R/E. On quantification of proteinuria, urine protein to creatinine ratio showed proteinuria of 1.3 g/g.

Given severe hypokalemia, the patient was admitted for parenteral and oral potassium replacement. The patient was given the daily replacement of 100 mEq/L parenteral and 40 mEq/L of oral potassium supplementation. His serum potassium was also monitored strictly. He was also prescribed antiemetics to control his vomiting which probably led to his low serum sodium, potassium, and chloride levels.

For the further workup of his hypokalemia, the following labs were obtained as shown in Table 2.

**Table 2:** Labs for the hypokalemia workup PCO_2_: partial pressure of carbon dioxide, HCO_3_: bicarbonate, ABGs: arterial blood gases.

Urine sodium	32 mEq/l (54-90 mEq/l)	24 hours urinary calcium (mg/24 hours)	28 (100-300)
Urine potassium	22.9 mEq/l (20-80 mEq/l)	Urine anion gap	28.9
Urine chloride	26 mEq/l (46-168 mEq/l)	ABGs	pH: 7.49, PCO_2_: 27.2 HCO_3_: 20.4

Table 2 shows low urine sodium, potassium, chloride and calcium, and positive urine anion gap. As the urine anion gap came out to be positive and urine pH was also alkalotic, a diagnosis of distal renal tubular acidosis was suspected. Findings of low urinary sodium, potassium, and chloride were considered to be secondary to vomiting-associated volume depletion. His ultrasound abdomen shows bilateral mildly raised normal-sized kidneys.

In order to identify the cause of his renal impairment and proteinuria, a detailed autoimmune workup was performed. Table 3 shows the list of investigations performed on this patient.

**Table 3:** Autoimmune workup ESR: erythrocyte sedimentation rate, ANA: antinuclear antibody, CPK: creatinine phosphokinase, ANCA: anti-nuclear cytoplasmic antibody, anti-GBM: anti-glomerular basement membrane antibody, anti-RNP: anti-ribonucleoprotein antibody, anti-dsDNA: anti-double-stranded DNA antibody, c3: complement 3.

ESR (mm/hour)	78	Anti-Smith/RNP antibody	83
ANA	Negative	Anti-PM-Scl antibody	60
c3	1.38	Anti-RNP antibody	34
CRP	37.26	Anti-dsDNA	Negative
ANCA profile	Negative	Anti-Ro antibody	Negative
Anti-GBM antibody	Negative	Anti-La antibody	Negative
CPK (U/L)	78		

As summarized in Table 3, the diagnosis of undifferentiated connective tissue disorder was made based on raised levels of ESR, CRP, anti-Smith/RNP antibody, anti-PM-Scl antibody, and anti-RNP antibody.

The possible cause of hypokalemia was considered to be distal RTA secondary to undifferentiated connective tissue disorder. Rheumatology consultation was taken and he was started on tablet hydroxychloroquine mycophenolate mofetil 1 g twice daily and tablet prednisolone six tablets twice daily.

Meanwhile, his renal biopsy was performed to determine the cause of proteinuria above 1 g/g and renal impairment which showed evidence of juxtaglomerular (JG) hyperplasia with hypergranulosis in six glomeruli consistent with a diagnosis of Bartter syndrome in the context of ongoing hypokalemia. There was moderate (35%) acute tubular necrosis as well. There was no evidence of interstitial fibrosis and tubular atrophy. Immunofluorescence showed mild to moderate intensity diffuse linear deposit of immunoglobulin A (IgA) and immunoglobulin G (IgG) along glomeruli and tubules.

**Figure 1:**
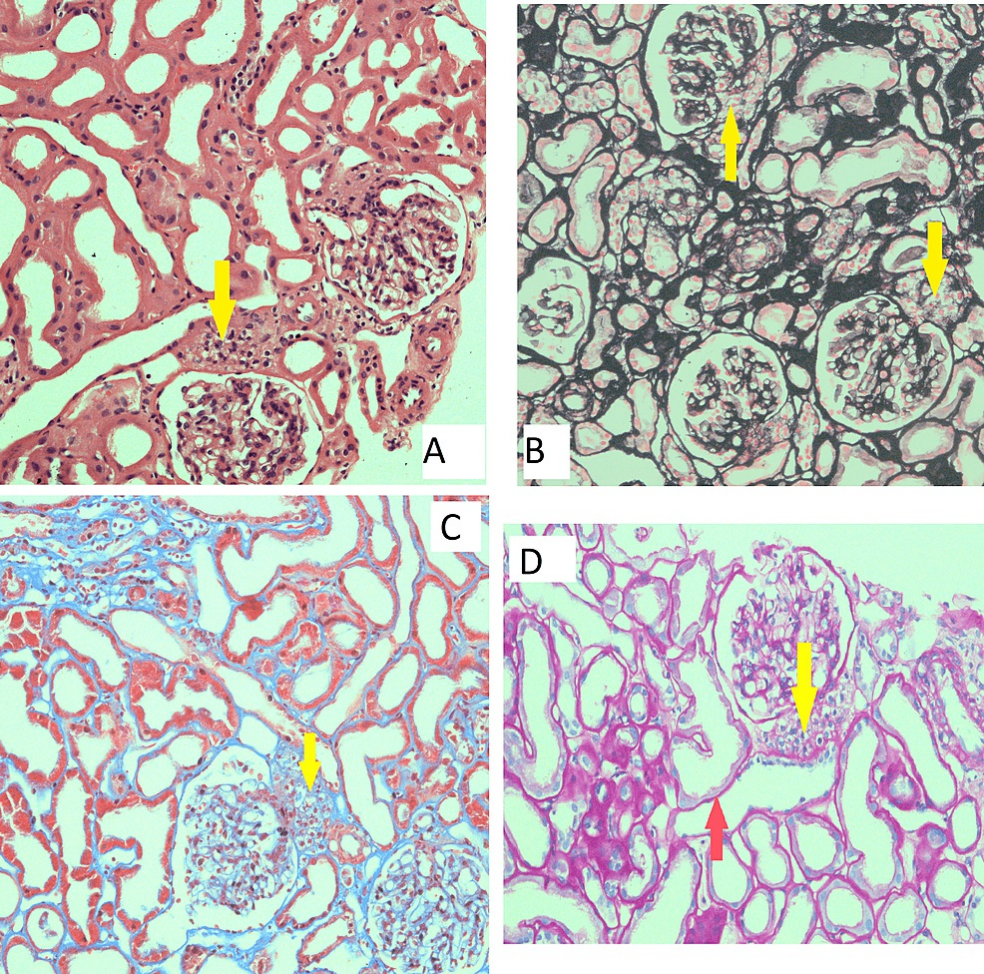
Light microscopic findings (40 × 10) (A) H and E stain, (B) Mathenamine Silver stain, (C) Trichome stain, and (D) PAS stain: JG cell hyperplasia (yellow arrow), acute tubular necrosis (red arrow).

Figure 1 shows light microscopic findings of this patient using different stains.

Light microscopic images using four different strains are shown in Figure 1. There is evidence of marked juxtaglomerular cells hyperplasia and acute tubular necrosis in these images.

**Figure 2:**
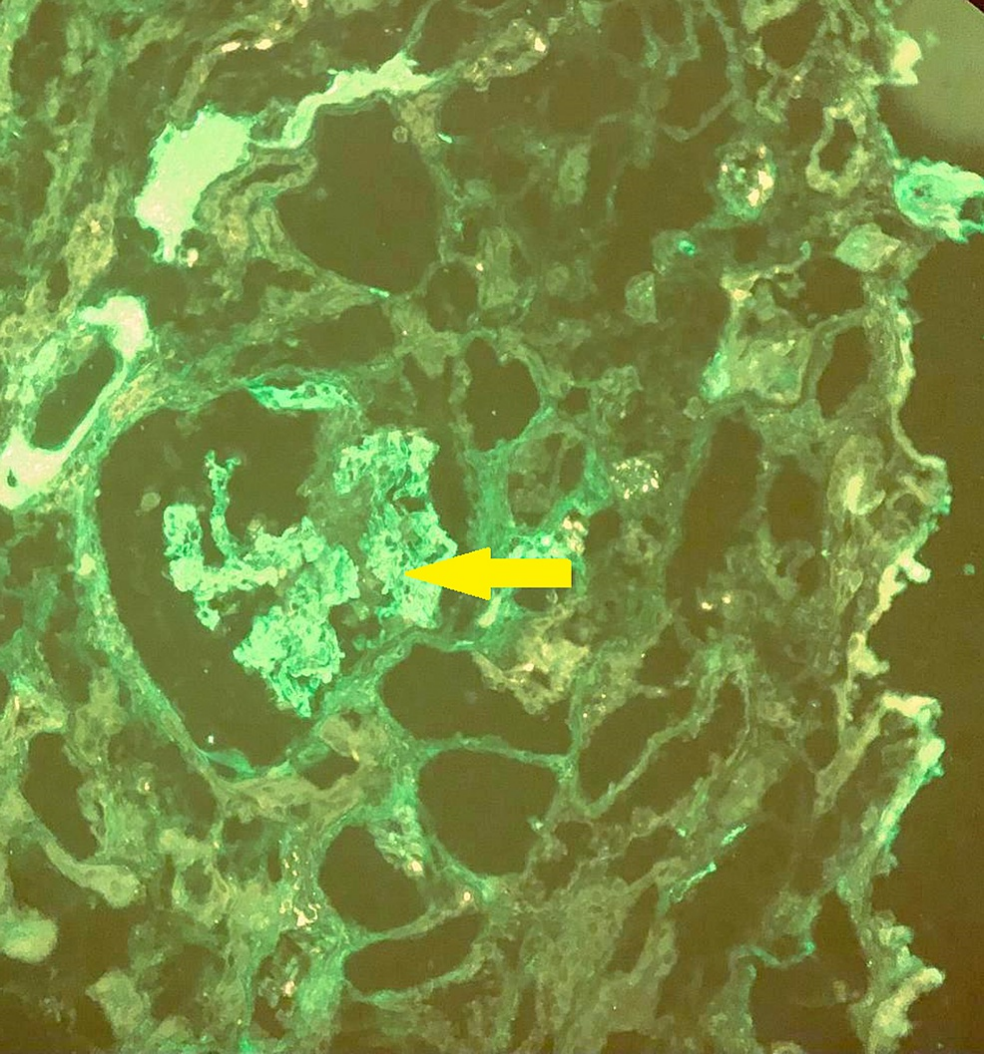
Immunofluorescence image (40 × 10) Evidence of linear IgA deposit (yellow arrow).

Figure 2 shows the immunofluorescence finding of this patient.

Figure 2 shows evidence of mild to moderate linear IgA deposits along glomeruli. Genetic testing could not be done due to non-availability at our setup. He was then started on tablet spironolactone for correction of his persistent hypokalemia. Non-steroidal anti-inflammatory drugs (NSAIDs) especially indomethacin were not started due to associated renal impairment.

After the follow-up of six months, there was sustained normalization of his serum potassium levels, reduction in proteinuria to around 700 mg/g, and improvement in serum creatinine from 3 to 1.5 mg/dl.

## Discussion

In summary, we report the case of a 21-year-old patient, who presented with lower limb weakness, marked hypokalemia, proteinuria, and renal impairment detected on laboratory evaluation. Undifferentiated connective tissue disorder was diagnosed due to positive autoimmune workup. The patient was given immunosuppressant therapy along with intravenous and oral potassium supplementation. Later on, the diagnosis of Bartter syndrome was supported by the presence of JG cell hyperplasia.

Bartter syndrome is a tubulopathy, with a predominant presentation during the antenatal period and childhood. As mentioned above, it has been classified into five types based on genetic mutations and age of presentation. Although types I, II, IV, and V are classified as antenatal Bartter syndrome, recently, adult-onset types II, IV, and V types of Bartter syndrome have been reported [6-9]. Our patient most probably had class III/classical Bartter syndrome. This type of Bartter syndrome usually presents during early childhood usually less than six years of age. However, the age of presentation and clinical severity is highly variable [10]. It is characterized by polyuria and propensity of dehydration, normal or high urinary calcium, and the absence of renal calculi [10,11]. Our patient also has no personal and family history of nephrolithiasis, nephrocalcinosis, hypocalcemia, and hypomagnesemia, therefore, likely ruling out all other types of Bartter syndrome and supporting the presence of class III Bartter syndrome.

However, the normalization of hypokalemia and renal functions in our patient after starting steroids and immunosuppressant therapy including mycophenolate mofetil, indicate that our patient might have acquired Bartter syndrome. But this cannot be said conclusively as genetic testing, to rule out/confirm the possibility of congenital Bartter syndrome was not done in our patient.

In one study, it was speculated that the existing antibodies from the autoimmune disease further enhanced the malfunctioning of the thick ascending limb and were responsible for clinical manifestations of the preexisting syndrome. It was also suggested that immunosuppressive treatment not only improved autoimmune disorders but also lead to the reduction of hypokalemia in Bartter syndrome [12]. The clinical course of our patient supports this proposition as the marked hypokalemia in our patient has responded after the introduction of immunosuppressive therapy including steroids and mycophenolate mofetil.

There are few case reports regarding the association of Bartter syndrome with autoimmune disorders. In a review of literature, five reports are published on Sjogren syndrome with associated Bartter [2], one on vasculitis with Bartter [12], one on Hashimoto thyroiditis with Bartter [4], and one on sarcoidosis with Bartter syndrome [13]. This is the first reported case of Bartter syndrome in an adult with undifferentiated connective tissue disorder.

Three antibodies were detected in our patient, namely anti-Smith RNP, anti-PM SCL antibody, and anti-RNP antibodies. Anti-Smith (Sm) antibody is a highly specific antibody for systemic lupus erythematosus (SLE). Anti-PM/Scl autoantibodies are found in polymyositis, dermatomyositis, systemic sclerosis (SSc), and systemic autoimmune disease overlap syndromes [14]. It is recommended that screening for antinuclear antibodies (ANA) is mandatory in cases of suspected SLE. Our patient had negative ANA levels, dsDNA, and normal complement levels, thus ruling out the possibility of SLE. The presence of normal creatinine kinase level also ruled out polymyositis.

It has been confirmed that Bartter syndrome is characterized by marked hypokalemia and metabolic alkalosis. According to many studies, the identification of high urinary chloride and calcium levels supports the diagnosis of Bartter syndrome. Our patient had atypical laboratory findings which included the absence of metabolic alkalosis and low urine chloride and calcium levels. Low urine chloride, at that time, might be associated with coexistent vomiting episodes in our patient. As mentioned, urinary calcium can be normal in class III Bartter syndrome [10] and in patients with coexistent renal impairment.

Possibility of vomiting as the cause of this clinical and laboratory presentation ruled out given the absence of metabolic alkalosis associated with vomiting; second, shorter duration and less frequent episodes of vomiting; third, is the persistence of marked hypokalemia in spite of settlement of vomiting, indicating tubulopathy.

According to one study [10], clinical features, laboratory findings, pathological evidence of renal biopsy, and genetic analysis are important in making an accurate diagnosis of Bartter syndrome. Our patient had typical clinical features (persistent marked hypokalemia with lower-limb paralysis episodes and hypotension) with atypical laboratory findings possibly due to associated vomiting, thus making the initial diagnosis of Bartter syndrome difficult. The most common and specific histopathological finding of Bartter syndrome is hyperplasia of the juxtaglomerular apparatus, with minimal or no glomerular or tubular abnormalities on renal biopsies. Our patient had juxtaglomerular hyperplasia with hypergranulosis, a characteristic finding of Bartter syndrome [15]. Juxtaglomerular hyperplasia can also be due to hypovolemic, hypokalemic disorders other than Bartter syndrome-like chronic chloride diarrhea, cystinosis, laxative abuse, and chronic vomiting [16]. Besides this, JG hyperplasia has also been associated with IgA nephropathy. However, the absence of mesangial expansion and granular IgA deposits rule out IgA nephropathy. The possibilities of these disorders have been ruled out in our patients. However, confirmation of Bartter syndrome could not be done due to the absence of genetic testing in our setup. It has, therefore, been proposed that in a patient with atypical presentation of hypokalemia, renal biopsy aids in making an accurate diagnosis.

To the best of our knowledge, almost all adult patients described so far have had well-preserved renal function [17]. Late manifestations in type III Bartter syndrome include proteinuria and impaired kidney function as a result of nephrocalcinosis. Our patient had renal impairment which might be due to persistent hypovolemia and hypotensive episodes leading to ischemic acute tubular necrosis (ATN), hypokalemia leading to tubular damage, and possible Dent disease with secondarily congenital tubular protein defect and associated tubular damage. Our patient did not have any nephrocalcinosis-associated renal impairment and focal segmental glomerulosclerosis (FSGS) [18], the two most common pathologies leading to renal impairment in Bartter syndrome.

Bartter syndrome is not classically associated with proteinuria [15]. In addition to these observations, as mentioned in few studies that a patient with coexistent Bartter syndrome-like features and low molecular weight proteinuria should prompt a clinician to search for Dent disease [19,20]. Dent disease is now considered a separate type of Bartter syndrome due to Bartter syndrome-like features in patients, described in previously few published studies [19,20]. In Dent disease, there is an abnormality of chloride/proton exchanger (ClC-5) located in the proximal tubule, the thick ascending loop of Henle, and the α-intercalated cells of collecting ducts. As a result of defects in the megalin and cubilin pathway, there is reduced proximal tubular reabsorption of low molecular weight proteins, phosphate, uric acid, and sodium leading to increased distal delivery leading to the hypovolemia-associated renin-angiotensin system and secondarily hypokalemia and metabolic alkalosis. Our patient might have isolated or associated with Dent disease as the patient had proteinuria with normal glomeruli suggesting tubular proteinuria. In addition to this, the patient also has hypophosphatemia, hematuria, and marked tubular damage in addition to juxtaglomerular hyperplasia indicating Dent disease with coexistent Bartter syndrome, respectively. However, due to the lack of availability of genetic testing, we cannot confirm whether our patient had isolated Bartter syndrome, isolated Dent disease with superimposed Bartter syndrome, or Dent disease with coexistent Bartter syndrome.

## Conclusions

To the best of our knowledge, this is the second case report of adulthood presentation of Bartter syndrome from Pakistan. It has several significant features which include a description of “adult-onset Bartter syndrome.” Besides this, it is the first case report that has described the possible link between Bartter syndrome and undifferentiated connective tissue disorder. In addition to this, it has given a clue about the possibility of Dent disease with Bartter-like phenotype in this patient, a proposition on which only a few case reports have been published till now. However, the major limitation of this case report is the non-availability of genetic studies in our setup. In spite of this, it will open doors for future researches.
